# Ultrafine Particle Metrics and Research Considerations: Review of the 2015 UFP Workshop

**DOI:** 10.3390/ijerph13111054

**Published:** 2016-10-28

**Authors:** Richard W. Baldauf, Robert B. Devlin, Peter Gehr, Robert Giannelli, Beth Hassett-Sipple, Heejung Jung, Giorgio Martini, Joseph McDonald, Jason D. Sacks, Katherine Walker

**Affiliations:** 1 Office of Research and Development, US Environmental Protection Agency, Research Triangle Park, NC 27711, USA; devlin.robert@epa.gov (R.B.D.); hassett-sipple.beth@epa.gov (B.H.-S.); mcdonald.joseph@epa.gov (J.M.); sacks.jason@epa.gov (J.D.S.); 2 Office of Transportation and Air Quality, US Environmental Protection Agency, Ann Arbor, MI 48105, USA; giannelli.bob@epa.gov; 3 Institute of Anatomy, University of Bern, 3000 Bern, Switzerland; gehr@ana.unibe.ch; 4 Department of Mechanical Engineering, University of California, Riverside, CA 92521, USA; heejung@engr.ucr.edu; 5 European Commission-Joint Research Centre, Ispra 21027, Italy; giorgio.martini@jrc.ec.europa.eu; 6 Health Effects Institute, Boston, MA 02110, USA; KWalker@healtheffects.org

## 1. Preface

In February 2015, the United States Environmental Protection Agency (EPA) sponsored a workshop in Research Triangle Park, NC, USA to review the current state of the science on emissions, air quality impacts, and health effects associated with exposures to ultrafine particles [1]. The workshop provided scientific presentations on the sources and trends of ultrafine particles (UFP) emissions and air quality concentrations, evidence of health effects associated with UFP exposure, metrics and indicators of UFP emissions, UFP measurement methods, control strategies, and policy considerations. This workshop brought together experts from around the world to share information and discuss future next steps on UFP research and policy. The following sections provide a summary of the presentations and discussions during this workshop, specifically highlighting the observations offered by individual speakers, summaries of the panel discussions, and potential opportunities to continue dialogue and enhance coordination and collaboration across multiple scientific disciplines.

## 2. Recent Reviews of the Scientific Evidence on UFPs

The workshop began with a summary of two recent reviews of the scientific literature on UFPs, one by the EPA as part of its 2009 particulate matter (PM) Integrated Science Assessment (ISA) [2] and the other by the Health Effects Institute (HEI) [3]. These reviews set the stage on how the weight of evidence is assessed during the National Ambient Air Quality Standard (NAAQS) review process in the United States of America (USA) by the EPA and the gaps identified in these reviews for the UFP literature that needed consideration in future research.

Jason Sacks of the EPA presented the overall conclusions of the 2009 EPA ISA, which formed the scientific foundation for the most recent review of the USA PM NAAQS that was completed in December 2012 [4]. As part of that review, the EPA assembled the available scientific evidence for UFPs and other PM size fractions to make causality determinations that reflect the overall weight of evidence for specific exposure durations (i.e., either short- or long-term exposure) and health or welfare effects (See Text Box 1 for the five-level hierarchy of causality determinations). The EPA’s approach to making causality determinations, which is supported by an independent panel of subject matter experts—the Clean Air Scientific Advisory Committee (CASAC)—draws upon the assessment and integration of evidence across scientific disciplines including atmospheric chemistry, exposure assessment, epidemiology, and experimental science (i.e., controlled human exposure and animal toxicological studies). The delineation between each level of the hierarchy is based on the ability to rule out with reasonable confidence chance, confounding, and other biases, but also takes into consideration dose and exposure concentrations along with the overall quality of the studies evaluated. The final causality determination for each exposure duration and health outcome also reflects consideration of input received from CASAC and the public during reviews of draft ISAs.

Text Box 1 **Hierarchy of causality determinations.**

Hierarchy of Causality:

- causal relationship
- likely to be a causal relationship
- suggestive of, but not sufficient to infer a causal relationship
- inadequate to infer a causal relationship
- not likely to be a causal relationship

For UFPs, the 2009 PM ISA concluded that the health evidence was suggestive of a causal relationship between short-term exposures and cardiovascular effects, with the largest body of evidence for changes in vasomotor function (the ability of blood vessels to expand and contract) and more limited evidence for changes in heart rhythm, along with cardiovascular-related hospital admissions and emergency department visits. It also concluded that there was evidence suggestive of a causal relationship between short-term exposure to UFPs and respiratory effects, including changes in lung function and pulmonary inflammation, with limited and inconsistent evidence for increases in emergency department visits and hospital admissions for respiratory-related events. Data were inadequate to draw conclusions regarding the relationship between short-term exposure to UFPs and additional health effects including premature mortality and central nervous system effects and between long-term exposure to UFPs and all of the health outcomes evaluated [2].

Several uncertainties and limitations in the health effects evidence contributed to the causality determinations presented in the ISA:

- Lack of data on UFP composition.
- Spatial/temporal variability in UFP concentrations.
- Incomplete information on the spatial/temporal evolution of UFP size distribution and composition.
- Limited number of controlled human exposure and toxicological studies that examined UFPs alone or in well-characterized combinations with other pollutants.
- Most studies involved exposures to older diesel exhaust which included a mixture of pollutants including other particle size fractions and gaseous co-pollutants, which contributed to uncertainty as to whether observed effects were due to UFPs, gaseous co-pollutants, or the combination of particles and gases.

The EPA recognized that the absence of a national network of UFP monitors to assess ambient concentrations in the United States of America has generally precluded the development of a national characterization of ambient UFP concentrations, including temporal and spatial patterns and trends, and therefore has provided limited information to support health studies [5]. This situation contributes to reliance on studies conducted on a smaller scale, and that use episodic and/or site-specific data sets to examine the relationship between UFP exposure and health effects. 

Based on full consideration of the scientific evidence presented in the ISA, the EPA concluded that the available scientific information was too limited to provide support for consideration of a distinct PM standard for UFPs. As a result, the EPA chose to retain PM_2.5_ as the indicator for fine particles in the 2012 PM NAAQS review [6].

Katherine Walker of the HEI summarized the objectives and findings of an HEI panel’s 2013 review of the scientific literature on ambient UFPs [3]. The HEI panel was tasked with clarifying the role UFPs play in the adverse health effects that have been associated with ambient air pollution, in particular with PM_2.5_. The report concluded that, despite some experimental support for concerns raised about the toxicity of UFPs related to their unique physical and chemical characteristics, the overall evidence from the last 20 years does not support a conclusion that exposures to ambient UFPs alone could account in substantial ways for the adverse health effects that have also been associated with other ambient air pollutants. A number of limitations in the experimental and epidemiological literature have made inferences about the specific role of UFPs challenging: differing definitions and measurements of UFP exposures, including use of near-road or traffic as proxies; limited consideration of confounding by co-pollutants; limited statistical power of small study designs; studies primarily examining only short-term UFP exposures; and limited coherence in the choice of and findings on various biological markers of health outcomes, among others.

Across the air pollution scientific community, it has been suggested that current scientific evidence has raised enough concern about exposure to UFPs that there is more that could be done to protect public health than the steps that have been taken to date. Katherine Walker challenged the group to articulate these concerns clearly in terms of scientific hypotheses and to identify the types of evidence that would be most informative to addressing them. Future data collection and research programs could be then designed to answer these questions as efficiently as possible. An ongoing challenge moving forward is how to better characterize the roles of UFPs distinct from and as contributors to the broader traffic mixture with which they are often associated. 

A broader research community effort is needed to help characterize the relationship between exposures to UFPs and their impacts on human health. To do so will require addressing the limitations of the research discussed above and during the workshop, and to consider carefully opportunities that current research methods offer. In particular, we need to move toward more consistent approaches to exposure assessment relevant to key health endpoints (e.g., the choice of UFP metrics, monitor location and types, measurement of covariates) that will facilitate assessment of the coherence of the scientific evidence. This synopsis of the UFP workshop captures a first step in that process.

## 3. Sources and Trends of UFPs

While UFPs can be emitted or formed from multiple sources, motor vehicles and other mobile sources often dominate emissions and exposures. This session, organized by Jorn Herner of the California Air Resources Board (CARB), Andrea Polidori of the South Coast Air Quality Management District (SCAQMD), Andreas Mayer of the Swiss Federal Institute of Technology Zurich, and Gayle Hagler of the EPA, provided background on UFP emissions and trends in ambient air. Jorn Herner of the CARB provided an overview of emission trends of UFPs from on-road mobile sources, specifically from heavy-duty trucks and light-duty vehicles. Comparing emissions from older vehicles to modern cars and trucks shows that emissions of UFP mass (UFPM) from both light-duty and heavy-duty vehicles have been reduced over the last several decades, often by more than 97 to 99 percent. However, the recent introduction of gasoline direct-injection (GDI) powered light-duty vehicles means appreciable amounts of UFPs can still be emitted, which led to a reduction in the Particulate Matter (PM) standard to 3 mg/mile in the USA and to 1 mg/mile in California in 2025. Emissions of UFP number (UFPN) showed very similar trends to UFPM with one notable exception: UFPN emissions from newer diesel vehicles with catalyzed diesel particle filters (DPF) operating under sustained heavy load, such as when driving on highways, were higher than emissions from older vehicles without a DPF. The DPFs remove almost all the traditional soot-based diesel particles, but under high temperatures, the catalyzed DPFs cause sulfur to be emitted as sulfate aerosol rather than sulfur dioxide (SO_2_) gas. While these particles have been measured near major freeways in California, toxicological assays indicate the particles have little toxicity. 

Although there is no formal definition for UFP, the vast majority of references use a cut point of particles of 100 nanometers (nm) or less in aerodynamic diameter (PM_0.1_). Herner noted that the vast majority of UFPs emitted from mobile sources are smaller than 100 nm. However, the UFPM peak is centered above 100 nm; therefore, he postulated that a definition of UFP using a 100 nm cut point may be appropriate for particle number but not for particle mass in the context of measuring emissions from mobile sources.

Michael Kleeman of the University of California at Davis provided an overview of fifteen years of measurements and modeling research in California. Transportation-related UFPN are found in higher concentrations near major roadways but concentrations quickly drop off to near background concentrations. Other sources may also be important on a regional scale, such as restaurants and wood burning. Modeling UFPs from near-source (e.g., roadside) to the community to farther downwind areas suggests changes in composition due to chemical transformations and an increasingly large fraction that cannot be apportioned back to specific sources. Also, there are significant diurnal and seasonal variations in both chemical composition and source profiles of UFPs. These factors appear to affect the toxicity of the particles, specifically the ability to cause oxidative stress, which was found to be higher in the summer and was hypothesized to be attributable to photochemical aging. The presentation also showed results from a preliminary effort to use exposure estimates from regional chemical transport models and health outcomes from the California Teachers Study to do epidemiological analysis [7]. The researchers used PM_0.1_ mass—which has a good correlation with ultrafine particle surface area (UFPSA)—as a metric and showed positive association between exposure and ischemic heart disease mortality. A more detailed study will expand the modeling for determining exposure and epidemiological analysis to further quantify the health effect specific to UFPM.

Andreas Mayer of the Swiss Federal Institute of Technology Zurich, presenting on behalf of Reinhard Zellner of the University of Duisburg-Essen, described ambient PM concentrations in Europe and strategies for research and policies to reduce PM-related health impacts. Zellner noted that important sources for UFPs in Europe include construction operations, mobile sources, agriculture, and wood burning during the winter season. Zellner suggested that other PM metrics may correlate better with health effects than the metrics used for existing air quality standards, PM_10_ or PM_2.5_. These new metrics could include: black carbon (BC), UFPM, UFPN, surface area, organic constituents (e.g., polycyclic aromatic hydrocarbons (PAHs), nitroaromatics), transition metals (e.g., copper, zinc), and endotoxins. However, the techniques needed to establish new metrics are only partly available at this time and will need further development, deployment, and field testing. He further acknowledged that health studies evaluating alternative particle metrics should include consideration of PM_2.5_, PM_10_ and other co-pollutants.

The session discussion included a debate on which metric of UFP is important to track and specifically for evaluating potential health effects—particularly particle number, surface area, or mass. Various researchers use different metrics and find them informative; however, comparing results across multiple studies can be difficult given the lack of consistency in the metrics used. Particle mass within the UFP fraction is much more challenging to measure than particle number. Ideally measurements would include a complete particle size distribution, which would allow using simple assumptions to determine all three. There was general agreement by participants that something larger than 100 nm, in the range of 200–500 nm, may be a more appropriate definition of UFP. Overall, consensus was lacking on the most informative and representative UFP metric to use in future studies.

## 4. Health Effects Evidence

Since the completion of the 2009 PM ISA as well as the 2013 HEI review on the health effects evidence of UFPs discussed above, there has been a growing body of literature further exploring health effects associated with exposure to UFPs. The UFP workshop highlighted this body of evidence in four presentations discussing epidemiological studies of short- and long-term exposure to UFPs as well as experimental evidence, principally from controlled human exposure studies in a session organized by Bob Devlin and Jason Sacks of the EPA, Peter Gehr of the University of Berne, and Linda Smith of the CARB. Annette Peters reported on three recent epidemiological studies evaluating short-term exposures to UFPs. The first was a multi-city time-series study conducted in five European cities as part of the European collaboration known as UFIREG (Ultrafine particles—an evidence-based contribution to the development of regional and European environmental health policy) [8]. The UFIREG project collected daily measurements of UFPN counts and PM_2.5_ to examine immediate and delayed associations with cardiopulmonary mortality and hospital admissions. They reported evidence of positive, but not significant, associations between short-term UFPN exposures and respiratory-related mortality for an UFPN exposure six days earlier. Respiratory-related mortality was not elevated for PM_2.5_. Cardiovascular-related mortality was not elevated for either UFPN or PM_2.5_. There was a positive association between respiratory-related hospital admissions and UFPN in two of the five cities; however, the association was more robust with PM_2.5_ than UFPNs. Dr. Peters also discussed an intervention study to assess measures taken to reduce air pollution during the Beijing Olympics [9]. Exposure measurements taken at central site monitors before, during and after the Olympics demonstrated a drop in UFPN, PM_10_, PM_2.5_ and NO_2_ during the Olympics. Panel studies conducted during this timeframe reported that decreases in multiple markers of inflammatory, cardiac, and hematological pathways were associated with both drops in UFPN and PM_2.5_. Since UFP are not homogeneously distributed within an air shed, and there are not robust monitoring networks for UFP, recent panel studies conducted in Germany and Canada assessed the relationship between health effects and short-term exposure to UFPN and PM_2.5_ using personal exposure measurements. These studies provided evidence of cardiac responses to UFPN in both single and co-pollutant models; however, associations with PM_2.5_ were equivalent or greater. Collectively, these short-term exposure studies provide some evidence of health effects attributed to UFP. When associations between UFP and PM_2.5_ and health effects were compared directly, in many instances they were more consistent and larger in magnitude for PM_2.5_. Peters suggested that measuring UFPs captures important properties of ambient aerosols that are not captured by PM_2.5_ measurements. She recommended that future research utilize multicenter studies of personal exposure to UFPs, with attention given to studies of appropriate power and design to reduce exposure misclassification, and to the joint measurement of exposures to black carbon and criteria pollutants in addition to UFPs.

Nino Künzli discussed long-term studies of the effects of UFP exposure. Epidemiology studies of the effects of long-term exposure rely heavily on characterizing spatial exposure gradients, which poses a challenge for UFP research, given that UFPN concentrations depend strongly on proximity to primary sources such as traffic. Adequate measurement networks are needed to estimate health effects associated with long-term exposures to UFPs, but such networks have largely been nonexistent until recently. Instead, studies have focused on characterizing health effects associated with residential distance to roadways. These studies generally report positive associations between proximity and cardiopulmonary effects. However, the results from these types of studies are complicated by the fact that people living along traffic corridors are exposed not only to UFPs, but also to other pollutants in the complex traffic mixture that have similar spatial patterns. Thus, the use of proximity to roadways as the exposure metric, rather than pollutant-specific measurements, is limiting in that such studies do not allow for the examination of the relationship between specific health effects and individual pollutants such as UFPs. A recent large study in Europe (European Study of Cohorts for Air Pollution Effects, ESCAPE) used land use regression modeling to estimate participants’ exposures to PM and its components and reported associations between cardiovascular effects and long-term exposure to traffic pollution; but UFPs were not part of the ESCAPE protocol. A recent study modeled estimates of UFP (PM_0.1_ mass) and showed that ischemic heart disease mortality was associated with PM_2.5_, UFPM, and secondary aerosols (SA) [10]. However, the study did not report whether health effects associated with UFP exposures were independent from the effects associated with PM_2.5_. The Swiss study on Air Pollution and Lung Disease in adults (SAPALDIA) study used measurements and modeling to assign long-term estimates of home outdoor exposure to UFPN as well as to NO_2_, PM_2.5_ and PM_10_ in four study areas [11]. The study reports high correlations between home outdoor concentrations of UFPN and PM mass, which explained 60% of the spatial variability of UFPN. Associations with health outcomes are currently being analyzed. Preliminary results indicate significant associations between home outdoor UFPN and carotid intima-media thickness, a marker of atherosclerosis. These analyses need to be further scrutinized with two-pollutant models with simultaneous adjustment for UFP and PM_10_ or PM_2.5_, which may be a challenge given the high correlation of UFPN with PM mass concentrations. Taken as a whole, Künzli concluded that the evidence from epidemiological studies is not yet sufficient to determine whether long-term UFP exposures can be associated with health outcomes to the same extent as has been observed in animal studies. He further stated the need to improve our understanding of the role of UFPs within the ambient mixture, specifically in evaluating possible high correlation of exposure to UFP with other pollutants and the extent to which the robust evidence of health effects associate with long-term PM exposure might be explained, in part, by UFPs.

James Samet discussed controlled human exposure studies to UFPs. These studies rely on devices capable of concentrating specific size fractions of PM from ambient air without any confounding gases or organic vapors. Additionally, controlled human exposure studies can examine the biological effects of very small UFPs (e.g., less than 30 nm), which dominate particle number readings, as well as larger particles in the 100–150 nm range, which dominate particle mass readings. These studies provide causality by directly assessing the biological effects caused by acute exposure to concentrated pure UFPs compared with baseline effects measured in the same subject following exposure to clean air. Studies conducted in three different locations have reported cardiopulmonary effects seen after exposure to UFP. Taken as a whole they demonstrate that concentrated UFPs can affect autonomic control of heart rhythm, endothelial cell function, and clotting/fibrinolysis pathways. Since these same pathways were shown to be altered in humans exposed to concentrated ambient PM_2.5_ particles, the studies provide some biological explanation for the mechanisms by which UFPs could plausibly cause adverse cardiovascular effects. Controlled human exposure studies in North Carolina also compared the relative toxicity of the three primary size fractions of concentrated PM (PM_2.5_, PM_10–2.5_, and UFPs) and concluded that, while effects could be observed following exposure to each size fraction, no one size fraction was more potent than the others [10]. 

Peter Gehr emphasized the importance of particle size because of the risk of translocation of solid UFPs from the lungs into the blood where they could attack a number of secondary organs such as the brain or heart. Gehr noted that there is some evidence that inhaled solid particles below approximately 300 nm can enter the bloodstream from the alveoli, translocate to organs, penetrate the blood-organ barrier and enter cells, cell organelles and even the cell nucleus. However, not all studies have been able to demonstrate such movement of UFPs. If true, this property of UFPs could provide biological plausibility to epidemiology studies that report associations between UFPN or proximity to roadways and adverse health effects. 

In summary, short-term epidemiological studies provide evidence that exposure to traffic pollution, which is enriched in UFPs, is associated with adverse cardiovascular outcomes. However, it is not clear whether these outcomes are more strongly associated with UFPs, or with some combination of other PM fractions or other pollutants that are co-located with UFP. Two long-term exposure epidemiological studies have used modeling approaches to estimate UFP exposures, but the relative absence of UFPN monitoring networks have not allowed for a comprehensive examination of long-term UFP exposures and adverse health outcomes in more locations. Controlled human exposure studies have shown that exposures to UFPs can cause biologic changes in several cardiopulmonary pathways, but similar to short-term exposure epidemiological studies, do not yet provide evidence to support the conclusion that UFPs are more potent than other PM size fractions. At the same time, concerns about translocation of UFP suggests that particle size may need to be considered in assessing the potential adverse effects of exposures to PM.

## 5. UFP Metrics and Indicators

The focus of this session, organized by Bob Giannelli, Tiffany Yelverton and Rich Baldauf of the EPA, and Heejung Jung of the University of California at Riverside, was to address questions relevant to UFP metrics and indicators of interest, such as:

- What are the UFP metrics that have been used to characterize potential emissions, air quality, exposure, and health effects?
- What are the strengths and limitations of these various metrics?
- Is there scientific support to advocate and/or prioritize a metric or group of metrics, either by study type or objective, for future research efforts?

The presentations reviewed the physical characteristics of PM emitted from the exhaust of internal combustion engines, fate in the atmosphere, the mechanisms for transport into the human body, and the strengths and limitations of possible UFP measurement metrics when considering potential human health impacts. These metrics included PM mass, PM number, PM size, Brunauer–Emmett–Teller (BET) surface area, “active” surface area, and particle reactivity which depends on chemical composition. Nearly all of the speakers agreed to a need for metric(s) that give information about how PM interacts the human body both during intake and transport through the body. Additional concerns were expressed regarding how measurement uncertainties for a UFP metric need to be minimized and the strong desire for any regulatory instrumentation to have minimal operational complexities. There was a general consensus that some kind of surface area metric could be considered and the definition of UFPs should be expanded to include particles greater than 100 nm. No consensus was identified for the actual upper bound value, although there was general agreement on the 200 nm to 500 nm size range.

David Kittelson from the University of Minnesota began this session by summarizing some key achievements in regulating PM engine emissions. In particular, he highlighted the reduction of sulfur in fuel facilitating the use of catalyzed exhaust filters for decreasing PM emissions and that the current EU number standards for solid particles above 23 nm are more stringent than either USA or EU mass standards. He noted that the EU standards effectively force the use of exhaust filters. He also indicated that the EU solid particle measurement method is very sensitive and can measure emissions at levels well below the noise level of gravimetric filter measurements. However, under some conditions, particles may nucleate and grow downstream of the volatile-particle remover (VPR) used in the EU method. Additionally, some advantages exist in using a catalytic stripper in place of a thermal denuder for measurement of solid particles. He suggested that low sooting engine or fuel technologies that meet both mass and number standards without exhaust filters may emit significant numbers of solid particles below 23 nm. 

Kittelson pointed out some ongoing issues associated with semi-volatile particles. Sulfur is typically stored on the DPF catalyst, and can pose a problem when catalyst temperatures reach approximately 350 to 370 °C. At these high temperatures and engine loads, the stored sulfur is released leading to nucleation and growth of sulfate particles and associated emission hotspots. He further reviewed sources and formation processes of engine particles and pointed out that emissions of Secondary Organic Aerosol (SOA) precursors are an ongoing concern that may have been underestimated. 

**Figure 1:**
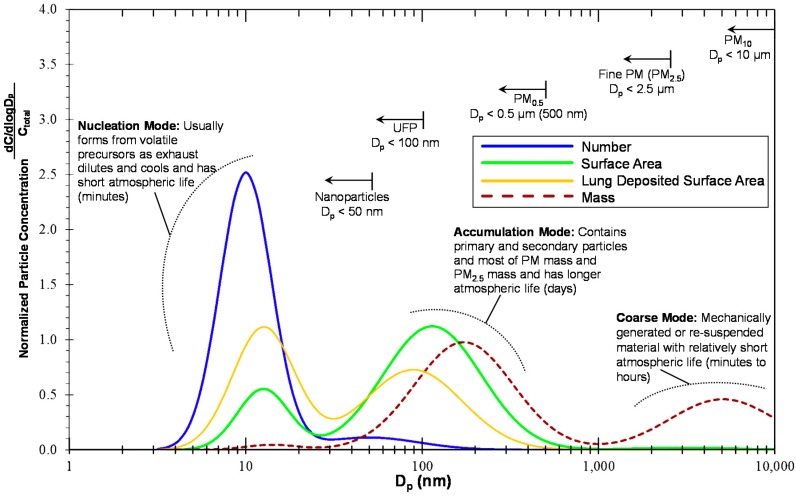
Tri-modal particle size distributions using different particle metrics (number, surface area, lung deposited surface area, and mass). For this figure, D_p_ is the particle diameter, UFP are ultrafine particles, and PM stands for particulate matter.

As discussed by Kittelson, engine aerosols are emitted in three size modes—nucleation, accumulation and coarse modes—and distinguished more by formation mechanism than by size. This tri-modal size distribution is shown for various particle metrics in Figure 1.

The nature of semi-volatile and non-volatile particles was also presented and Kittelson concluded that nucleation mode particles are mostly semi-volatile. Most of the exhaust particle mass and particle number exist in accumulation and nucleation mode, respectively; therefore, he argued that the arbitrary definition of UFPs (particle diameter (D_p_) < 100 nm) does not correlate well with either total particle number or mass. He suggested to re-examine the definition of nanoparticles and ultrafine particles, such that:

- Nanoparticles, D_p_ < 30 nm; likely to be highly correlated with total particle number.
- Ultrafine particles, D_p_ < 500 nm; capturing accumulation mode mass.

Diffusion chargers were suggested as inexpensive instruments for measuring “active” or “lung deposited” surface area which can include both nucleation and accumulation modes in nearly equal weight. In addition, these chargers are low cost, which might encourage wide deployment for epidemiological studies.

Imad Khalek from the Southwest Research Institute followed with an overview of the current state of PM regulations in the USA and the EU. In the USA, due to the use of particle filters, PM emissions from heavy-duty highway vehicles are 90% below the 0.01 g/bhp-hr USA regulatory standard. For light-duty vehicles in 2017, the EU will have a stringent solid particle number limit of 6 × 10^11^ particles/km, which is equivalent to ~0.5 mg/km (~0.8 mg/mile). This is more stringent than the California Air Resources Board (CARB) low emission vehicle (LEV) III regulations of 1 mg/mile when fully implemented in 2025 (CARB LEV III 2025), because the EU standard essentially forces the use of gasoline particulate filters for GDI vehicles. As manufacturers increasingly use GDI for fuel economy gains, the PM emissions will increase relative to port fuel injected (PFI) engines, especially immediately following the engine cold start.

Significant improvements in vehicle exhaust PM mass measurement methods have been made to improve repeatability and minimize artifacts. The method gives sufficient measurement confidence for a 1 mg/mile standard of CARB LEV III 2025 light-duty vehicle emission standard, and suggests that it is also feasible to lower the heavy-duty PM mass standard from the current 10 mg/bhp-hr to 3 mg/bhp-hr. While solid particle number measurement is a very sensitive and well-developed technology, it excludes semi-volatile particles and has not yet been established to have a relationship with adverse health effects. The total particle number method includes semi-volatile particles as well but the method is highly sensitive to dilution conditions. Khalek suggested measurement of particle size distributions as the only way to capture size and other important metrics such as mass, number, and surface area. Additionally, he noted that significant effort will be required to further develop the measurement protocol including the appropriate calibration methods. Measurement of particle precursor mass is another possible alternative metric; however, the method does not contain particle size information which is important to lung deposition. Also, significant development is needed to establish a particle size distribution method. 

Three studies were presented by Alfred Wiedensohler from the Leibniz Institute for Tropospheric Research specifically evaluating the reduction of black carbon emissions from vehicles and its influence on UFP concentrations in ambient air quality. These studies included measurements of particle number and PM_10_ mass: (1) a low emission zone in Leipzig, Germany, required due to past exceedances of the EU PM_10_ standard; (2) the German Ultrafine Aerosol Network (GUAN); and (3) a measurement of PM on a census day in La Paz, Bolivia. 

A low emission zone (LEZ) was established as a major clean air action plan in Leipzig, Germany. Traffic-related emissions accounted for 5% to 10% of the PM_10_ mass concentrations while regional background PM accounted for more than 60% of the PM_10_ mass. Regardless of installation of the LEZ, exceedance days for PM_10_ were not reduced significantly. However, data from the past five years showed BC concentrations (measured with multi-angle absorption photometer or MAAP) were reduced significantly at all measurement sites following implementation of the LEZ (only Euro 4 equivalent diesel cars, light- and heavy-duty vehicles, and gasoline cars with catalyst were allowed within the central/low emission zone of Leipzig) and other efforts related to the clean air program. 

The GUAN aims to provide data to study the link between the number of particles from 60 to 300 nm (PN_60–300 nm_), as size differentiated number counts, to follow reduction targets of BC and to estimate the health risk associated with BC and UFP. GUAN networks are in many locations and the data clearly show correlation between BC and PN_60–300 nm_. Both BC and PN_60–300 nm_ shows clear reduction in concentrations annually.

Finally, Wiedensohler presented a study comparing BC and PN_60–300 nm_ on a census day in La Paz, Bolivia, a day when everyone must stay home so no vehicle traffic occurs, with a typical weekday. This study showed a marked reduction in both pollutants as well as carbon monoxide (CO) on the census day, indicating the important contribution of vehicular traffic to the mixture of air pollution in La Paz.

Issues related to modeling UFPs were discussed by Max Zhang from the Cornell University. He discussed regional nucleation events during the 2008 Beijing Olympic and showed that new particle formation (evidenced by appearance of high particle number concentrations) generally occurs when PM_2.5_ mass concentrations are low. Particle size distributions were measured on the rooftop of a six-story building near the Olympics venues. This inverse correlation followed the physics of particle formation in terms of super saturation ratio for nucleation and available particle surface area for condensation. These types of regional nucleation events have been observed in many other USA urban areas such as Pittsburgh, St. Louis, and Atlanta. Zhang claimed that nucleation mode particles from combustion sources can be better modeled and predicted taking into account the dilution rate as well as the dilution ratio. These characteristics provide the ability to link laboratory measurements of vehicle exhaust particle distributions with those from on-road chase experiments. He suggested integrated experimental and modeling approaches. In a demonstration of the method to unify laboratory and on-road measurements, he presented results from computational fluid dynamics (CFD) coupled with PM chemistry that provides an understanding of the intricacies of dilution systems and how they can differ and agree. Modeling has the additional advantage of outputting results in any desired metric, such as mass, number and surface area, once good input parameters such as emission inventory, and comparison between model prediction and experimental results are established. Current UFP studies are focused on traffic-related emissions; however, emerging sources such as distributed generators and biomass combustion are potentially of concern.

Günter Oberdörster of the University of Rochester focused on the toxicological effects associated with UFP exposures. In his view, the recent International Agency for Research on Cancer (IARC) decision to categorize ambient air pollution as a Group 1 human carcinogen is a strong justification to regulate anthropogenic emissions more strictly with UFP as one precursor of PM_2.5_.

Oberdörster considers dose as the key parameter for toxicology. Physico-chemical properties of UFPs such as size, solubility and reactivity are relevant to toxicology. UFPs have high percentages of surface molecules per mass and BET surface area of particles shows good correlation with the ability to generate reactive oxidative species (ROS). Surface reactivity, like ROS generation ability, can be used as a screening tool to categorize UFPs by reactivity. In his experiments, particle mass or number have not correlated well with ROS generation by particles from ambient air. The main deposition mechanism for UFPs is by diffusion where all regions in the lung can be targeted. UFPs are small enough to be able to contribute to translocation across the cell barrier. Once particles translocate into the bloodstream they can reach any organ in the body including the brain. Of special interest is the high deposition efficiency by diffusion of UFP in the nasal region, particularly for particles below ~15 nm, because of translocation via axons of olfactory neurons into the olfactory bulb of the central nervous system. The gastrointestinal tract (for all UFP sizes) and kidney (for particles ≤6 nm) are thought to be the major excretory organs for elimination of particles in the body. Determining the best metrics representing health effects can be difficult due to diverse physico-chemical properties of UFPs, which can lead to different toxicity. However, a particle surface area-related metric seems to be best correlated with biological/toxicological activity.

Oberdörster stated that particle surface area and ROS generation potential are good measures for evaluating dose-response while particle number (preferable) and mass (often too low) concentrations are useful metrics for characterizing exposure. A universal UFP standard (targeting all UFP) based on any one-exposure metric may not make sense because differences in physico-chemical properties (source specific UFP; anthropogenic, natural) give rise to significant differences in toxicity. Oberdörster proposed consideration for source-specific standards based on:

- Identification of sources that emit the most reactive UFPs (based on comparative hazard identification using proper dose metric).
- Establishment of a number emission standard, based on source-specific UFP risk assessment (rather than for all UFP).
- Characterization of number concentration of emitted UFP (consider UFP down to <10 nm).
- Consideration of co-pollutants (other particle size fractions, gases).

A general discussion followed these presentations primarily focused on the strengths and limitations of various metrics. A number of challenges to utilizing UFPN to evaluate potential health effects were noted. Herner found an inverse correlation between particle number and health effects from his source testing with a diesel particulate filter-equipped vehicle. Zhang also showed an inverse correlation between PM_2.5_ and particle number concentrations in nucleation events. Künzli, however, pointed out that they had observed a high correlation between UFPN and PM_10_ in the SAPALDIA study, and that regional nucleation events were rarely observed in urban areas in Europe. Particle surface area was viewed as a good alternative metric, although opinions varied on whether BET or active surface area was most suitable and practical. Kleeman mentioned that PM_0.1_ mass has a good correlation with particle surface area for particles less than 0.1 µm. Several speakers suggested defining a new upper-limit cut-off point for UFPN around 200 to 500 nm in order to include all traffic or combustion-generated particles.

## 6. Instruments and Methods

This session, organized by Andreas Mayer of ETH-Zurich, Giorgio Martini of the European Union Joint Research Council, Andrea Polidori of SCAQMD, Greg Smallwood of the National Research Council Canada, Heejung Jung of University California at Riverside, Joe McDonald of EPA, Michael Breen of EPA, and Gayle Hagler of EPA, contained presentations addressing questions relevant to UFP instrumentation and measurement methods, including:

- What techniques/instruments are available to measure/characterize UFPs in source emissions, ambient air, and personal exposures? What are the strengths and limitations of each of these techniques?
- Which techniques may be most useful for consideration in epidemiological studies and why?
- What modeling techniques can be used for measurement methods for emissions, ambient air and indoor air evaluation, and what are the strengths and limitations of the available models?
- Evaluation and relevance of techniques and methods for each category (emissions, ambient air, exposure, health).
- Is there scientific support to advocate and/or prioritize techniques and methods, either by category or across all categories?

**Figure 2:**
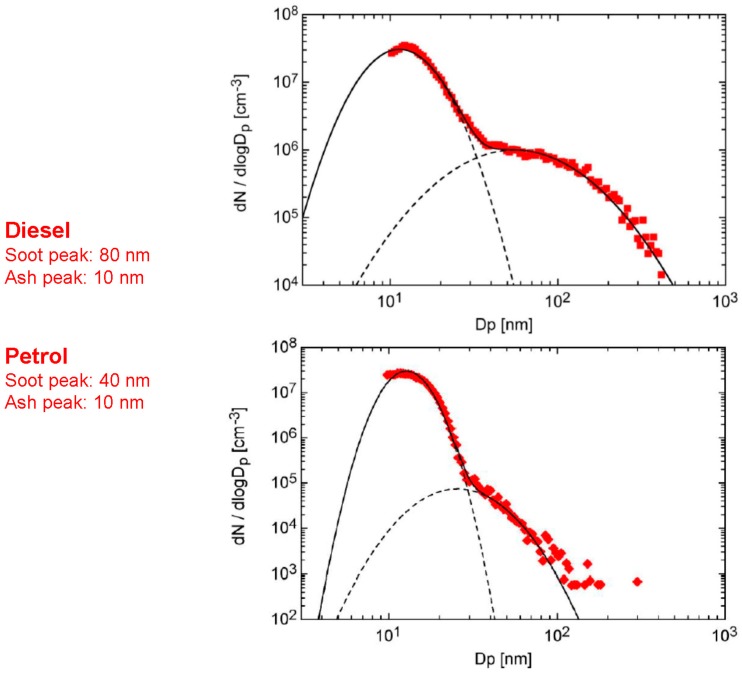
Internal combustion engine particle emissions; diesel (**top**) and petrol or gasoline (**bottom**).

Andreas Mayer of the TTM gave an additional presentation on behalf of Heinz Burtscher, from the University of Applied Sciences and Arts Northwestern Switzerland providing an overview of ambient UFP field instruments based on the diffusion charging principle. Mayer noted the importance of measuring UFPN and how this metric can provide more information than just ambient PM mass. Particles emitted by diesel and gasoline engines have a number peak for soot particles around 40 and 80 nm, respectively (Figure 2). Also in ambient air, particle size distributions indicate a number peak around 100 nm while the use of mass parameters like PM_2.5_ or PM_10_ are dominated by larger particles which have lower number counts [12]. Furthermore, fine particulate mass (PM_2.5_) and particle number are not always correlated due to a number of factors, including scavenging and small particle coagulation onto larger particles when number concentrations are high. Low mass concentrations of PM_2.5_ can correspond to high particle number concentrations. While particle mass concentrations have relatively homogeneous spatial distributions, UFPN varies strongly spatially, particularly with distance from a road. This variability in UFPN has presented challenges in evaluating potential human health effects, although Mayer suggested that an approach similar to those used in occupational settings may be appropriate, such as a local “not to exceed” concentration limit (10,000 particle/cm^3^ was provided as an example) for public places like schools and community centers. This approach would warrant the need for strategies to reduce potential public health impacts that may not be supported by the use of urban-scale measurements.

Mayer discussed an effort to develop portable instruments based on diffusion charging principles to measure UFPN that could be used to support this approach. This technique electrically charges particles by diffusion of ions with the resulting current measured by an electrometer. This instrument also provides particle size distributions. The equivalent mass can be calculated by multiplying the particle effective density profile and the particle surface area (either equivalent mobility, spherical particle surface area or active particle surface area). Another type of instrument discussed uses a corona wire with pulsated current to detect image charges. With this instrument, the particles remain available after measurement for analysis since no filter is needed to measure the current. This instrument can be miniaturized and powered by batteries for personal monitoring. Diffusion charging based instruments can also be used to collect information on the nature of the particles by use of thermal pre-treating before the measurement.

Mayer concluded that diffusion charging-based techniques offer a wide range of possibilities for particle characterization. These techniques cannot replace other more sophisticated techniques typically used in laboratories, but can be ideal for ambient and occupational applications, providing additional information on particle characteristics. Since these instruments are commercially available, data exists on instrument performance and reliability.

Jon Andersson of Ricardo, UK provided an overview of the European particle number legislation that led to the development of the Particle Measurement Program (PMP). Andersson discussed the scientific and political context that led to the PMP to improve particulate measurements while promoting the use of Diesel Particulate Filters (DPFs) in diesel engines in Europe. Although questions remained about the effect of particle size and number on human health at the time of the PMP, the “precautionary principle” provided a justification for the removal of diesel particles with a DPF. This technology had also been demonstrated to be feasible and effective in light-duty diesel vehicles (LDVs). However, due to the poor repeatability of gravimetric measurements at the low levels of post-DPF emissions, an alternative PM method was required. With an initiative by the UK Government, and with strong political backing from France, Germany, Holland and Sweden, a working group was established under the United Nation’s Global Party on Pollution and Energy (GRPE) with the objective of developing alternative metrics with increased sensitivity for solid ultrafine particles from heavy-duty and light-duty vehicles. 

The mass and particle counting measurement system developed within the PMP defined the solid particle measured as:

- Non-volatile (only particles counted after exposure to 350 °C for 0.25–0.4 s).
- Nominal minimum size (particle cutpoint, D_50_ = 23 nm).
- Nominal maximum size (D_50_ = 2.5 μm).

Andersson noted that calibration of the equipment has been a significant challenge. He also noted that UFPN limits in the European Union of 6 × 10^11^ particles/km have been set to mandate DPFs, currently for light-duty diesel vehicles (LDDVs) and for GDI vehicles in 2017. There is no direct relationship between PM and PN in European regulations but PM remains part of the regulation mainly to control volatile material. The alignment of GDI emission standards with LDDVs could lead to a widespread use of gasoline particle filters (GPF). In addition, a particle number limit may be introduced soon for off-road engines that could force the use of DPFs. The PMP working group continues to address the following main tasks:

- Determine for both heavy- and light-duty vehicles the need to revise the lower particle size D_50_ cut-point of 23 nm especially taking into consideration the extension of the particle number limit to gasoline engines.
- Investigate exhaust particle emissions of port-fuel injection vehicles, currently exempt from Euro 6 UFPN emission limits, in particular under fuel rich operation conditions (e.g., cold start, high load).
- Update existing calibration guidelines especially for new cut-point. Reducing the cut-point to 10 nm could be achieved even with the current set-up but the calibration procedure will become more difficult.

Alla Zelenyuk from the Pacific Northwest National Lab presented the multidimensional particle characterization system, which allows measurement of many of the relevant PM properties with very high sensitivity and precision as well as temporal resolution. This system has a goal of understanding formation, chemical and physical properties of PM, including the evolution of small particles. The system is based on a single particle laser-ablation time-of-flight mass spectrometer (different versions, SPLAT, SPLAT II, miniSPLAT). These systems are both laboratory and field deployable and can characterize particles from 50 nm to 3 μm. The system can measure the size of particles with a precision of 0.5% at a rate up to 5000 particles/s, and uses infrared and ultraviolet techniques for evaporation and ion formation to generate data on particle composition at a rate of up to 100 particles/s. These systems have been used successfully to perform in situ measurements of many different particle characteristics including size, number, composition, density, shape, morphology, phase, chemical diffusivity, viscosity, interaction with water vapor, reactivity, and evaporation kinetics. Novel approaches to analyze, classify and visualize the data collected had to be developed and this is an essential aspect to exploit the many possibilities offered by this technique.

An example of the application of these techniques is the investigation of the properties of SOAs. These particles play an important role in air quality but for many years available atmospheric models were not able to predict SOA formation. The main issue was the fact that all models relied on the assumptions that SOA particles were well-mixed low viscosity solutions and maintained equilibrium with the gas-phase by rapid mixing in the condensed phase with evaporation and condensation. Recent studies using the multidimensional characterization approach demonstrated that these assumptions were wrong and that SOA particles must be viscous semi-solid. These studies showed also that there is a synergetic effect between PAHs and SOA since PAHs trapped inside the SOA particles slow down SOA evaporation and increase SOA yield and lifetime. This can explain the long-range transport of toxic compounds like PAHs and other persistent pollutants. In conclusion, a new SOA paradigm has been developed: particles are semi-solid, nearly non-volatile and trap organic material during formation. The particles are not in equilibrium with the gas phase.

This approach can also be used for exhaust particles. Under the majority of operating conditions, particles from diesel engines are fractal soot particles with a composition dominated by elemental carbon. However other particles with different physical characteristics and composition were identified. Particles from Direct Injection Spark Ignition (DISI) engines were also investigated. Huge differences were noticed depending on the load of the engine. At low load conditions particles are dominated by compact organic particles with small contribution of fractal particles. At high loads, particles mainly consist of fractal soot agglomerates containing 40%–45% organic material. Moreover, under some conditions two distinct modes can be identified with a marked difference in the size of the primary spherules. The smaller particles seem to be generated in fuel rich regions while the bigger particles seem more linked to wall/piston fuel impingement. 

The presentation by Hans-Georg Horn, TSI GmbH provided information on the efforts taking place in Europe for the development of a standard method for ambient particle number monitoring. PM, PN, and lung deposited particle surface area (LDSA) are all measures for which reliable instrumentation exists. All of these have advantages and disadvantages. For example, PN is a very sensitive measure of UFPN and shows good correlation with combustion sources and traffic emissions. However, UFPN based studies on the effect on human health are still lacking. On the other hand, PM_10_ and PM_2.5_ mass is poorly sensitive to UFP number and mass, although they are the basis for many epidemiological and toxicity studies. 

The effect of the low emission zone in Leipzig on particle number concentration was shown as an example of what can be noticed by using UFPN that could not be detected using mass indicators. For ambient UFPN monitoring, the technologies that could be applied are: Condensation Particle Counter (CPC), diffusion charger and electrometer, diffusion charger and electrometer with diffusion battery stage and two electrometers (e.g., DiscMini). On the basis of the characteristics of these available technologies and also in consideration that the CPC is already used in exhaust emission regulation and that the reliability of these systems is sufficiently proven, the CEN TC 264/WG 32 committee has chosen condensation particle counters to define the standard method for particle number concentration measurement in ambient air. The decision to develop such standards was taken to overcome the problem that no regulation would be introduced for ambient air PN monitoring without a standard method and, at the same time, air quality monitoring networks would not include PN measurements without a regulation. Standardizing CPCs for air quality monitoring is important in order to achieve good accuracy, repeatability, and reproducibility of the measurements. This requires the definition and standardization of a number of parameters like lower particle size detection efficiency curve, working fluid, tolerance for linearity of response to concentration changes and changes in ambient conditions. It is also important that traceability of measurements is established by means of well-defined measurement protocols and the CPC itself must be traceably calibrated. Moreover, the sampling system is an important part of the particle number concentration measurement system and must be optimized for the specific purpose. 

A comparison between the requirements defined in the CEN TC 264/WG 32 for ambient air monitoring and the recommendations for CPCs used in roadside measurements as part of the USA EPA’s new near-road monitoring network was presented by Horn. Some of the requirements are quite similar but there are also significant differences. The measurement of the size distribution was briefly discussed. The use of particle size distribution for regulatory purposes is much more complicated than the particle number concentration and it would be even more complicated deriving any limit value. 

The objective of the presentation by Bob Giannelli, USA EPA was to provide an overview of the activities at USA EPA on particulate emissions, including UFP. Considering that further reductions in emission standards may approach levels at which gravimetric measurement methods are limited, there is an effort to continue developing expertise in measurement and sampling techniques for PM that includes size, number, surface area, chemistry, and other pertinent metrics to determine sources of variability and parameters that enable control over variability to develop analytical tools for a better understanding of PM transport in sampling systems. The final goal is to develop UFPN measurement techniques for mobile sources with the same accuracy as for PM mass. One of the projects is related to the control of non-volatile PM emissions from aircraft engines that are quite different from PM emissions from road engines. Particle number is measured using the PMP method with the addition of a catalytic stripper to get to smaller sizes. A particle loss estimation method had to be developed as a consequence of the long sampling lines needed in the engine test stands.

This USA EPA PM Number Initiative has the objective of understanding and modelling the transport mechanisms in sampling systems with the final goal of understanding and controlling measurement variability. Model development is underway and the intention is to validate it initially with simple aerosols and then with engine exhaust by means of engine and vehicle testing with constant volume sampling (CVS) systems. There is also the intention to continue improvements in laboratory capabilities (chemical, mass and size analyzers, sampling systems, particle sources, multi-physics software), to continue the modelling work starting from simple aerosols and gradually extending to more complicated aerosols. Longer term goals include extending these evaluations to vehicle exhaust and CVS systems as well as to develop a “transfer function” for atmospheric modelling. 

One of the main questions discussed during this session was which technique might be most useful for consideration in epidemiological studies and why. Based on the presentations and subsequent discussion, the answer remained elusive for most participants. Advantages and disadvantages of several possible metrics and methods have been highlighted, with many noting that just one metric may not be sufficient. The anti-correlation of PM_2.5_ and UFPN in ambient air provides a good example of how mass and particle number information can be complementary. 

This session highlighted the wide range of instruments and measurement methods that allows detailed analysis of physical/chemical properties of particles; however, there is no one instrument that provides all metrics. Thus, instrumentation choice will depend on the application. A few examples of applications and key points made by presenters and discussants included:

### 6.1. Fundamental Research on Physical/Chemical Characteristics of UFP

There is no limit to the complexity of the instrument and the number of different instruments that can be deployed. The multidimensional characterization of particles illustrated by Zelenyuk is an excellent example. Many particle properties can be investigated at the same time and a lot of information can be obtained on many different aspects. One of the challenges of these approaches is to find the best way to analyse and visualize the large amount of data collected. As shown, these systems are not confined to the laboratory environment but can be used in the field.

### 6.2. Ambient Air Monitoring

A few different instruments could be used for ambient measurements. In both the USA and Europe, the tendency is to prefer CPCs compared to other instruments. It has been pointed out that standardization of the CPCs and measurement protocols is a pre-requisite to ensure good accuracy, repeatability and reproducibility. An effort is taking place in Europe to standardize the requirements for CPCs for ambient air monitoring. The roadside particle measurements being carried out in the USA as part of the near-road monitoring network are part of a learning process and data collected will be accessible.

### 6.3. Local and Personal Monitoring

If the objective is to obtain the particle active surface area concentration in specific places (e.g., buses, workplaces, schools) or to assess the personal exposure to particles, instruments based on diffusion charging and electrometers could represent the right compromise in terms of accuracy, ease of use, and instrument size. These instruments can even be miniaturized to produce wearable devices for personal monitoring. 

### 6.4. Measurement of Solid Particle Number for Certification of Engines and Vehicles

Instrument and measurement procedures for regulatory purposes have to fulfil a number of specific requirements such as accuracy, repeatability and reproducibility. The particle counting procedure developed within the PMP required the combination of political backing (mandating DPFs) and the technical requirements mentioned above. Proper pre-conditioning of the engine before the measurement has also been mentioned as an important aspect influencing the variability of the tests but in the experience of the PMP, it was not possible to reach an agreement on this point.

Calibration is a challenge especially when the particle size is very small. The use of different calibration aerosols, particle sources and particle materials can lead to significant discrepancies even between instruments using the same principle. Furthermore, taking into account losses in the sampling systems is another scientific and practical challenge. It is recommended that a calibration procedure as simple as possible should be a main objective when developing procedures for regulatory purposes. Overall, techniques, instruments, procedures and calibration must be carefully optimized depending on the intended use and application.

## 7. UFP Control Strategies

The session discussing control strategies included two presentations related to mobile source controls and one presentation on stationary source controls, and was organized by Joe McDonald of the EPA and Michael Benjamin of the CARB. 

### 7.1. Mobile Sources—Diesel UFP Control

The presentation by Joe Kubsh of the Manufacturers of Emission Controls Association focused on the use of catalytic exhaust emissions control systems and evaporative emissions control systems to control mobile source emissions of both primary and secondary particulate matter and UFP emissions from light-duty, heavy-duty and nonroad vehicles and equipment. Dr. Kubsh summarized the application of DPFs with wall-flow substrates into millions of light-duty and heavy-duty diesel applications worldwide. Such systems provide in excess of 90% reductions in particulate matter mass emissions and in excess of 99% reductions in solid particle number emissions. Research funded by the USA Department of Energy (DOE) and HEI has shown that diesel applications equipped with catalyzed DPFs also remove nearly all emissions contributing to secondary organic aerosol formation and significantly reduce hydrocarbon emissions, air toxic emissions and metallic ash particle emissions from 2010 and later heavy-duty diesel engines in the USA [13]. The catalytic emission control systems used with 2010 and later heavy-duty diesel engines in the USA combine Selective Catalytic Reduction (SCR) for NOx control with catalyzed DPFs for PM control. These systems rely primarily on the use of passive PM regeneration via NO_2_ oxidation of soot trapped within the DPF instead of relying on active DPF regeneration. This further reduces PM emissions that are associated with active regeneration events and reduces fuel consumption compared to the 2007–2009 engines that primarily used active DPF regeneration. More recently, DPFs have begun to directly incorporate SCR catalyst coatings onto wall-flow substrates to create “four-way” diesel catalyst systems that simultaneously control HC, CO, NOx and PM emissions. Integration of these functions onto a single substrate can allow reductions in total system volume, backpressure and cost in diesel emissions control systems. Emissions control retrofit programs have also resulted in more than 100,000 DPF retrofits of older heavy-duty diesel bus and truck applications over the last decade in the USA, with California retrofits accounting for more than half.

Dr. Kubsh characterized USA Tier 4 Final Nonroad and EU Stage IV Off-road diesel emissions regulations as not setting stringency equivalent to that of on-highway diesel standards for NOx emissions or PM mass emissions. More than half of engine families in the 130 kW to 560 kW range have been certified to USA Tier 4 Final Nonroad standards without the use of DPFs [11]. In Europe, Stage V Off-road diesel standards are now under consideration with a new limit on particle number emissions in order to force the use of DPFs into these applications.

### 7.2. Mobile Sources—UFP Control from Light-Duty Gasoline Vehicles

In 2001, the California Zero Emission Vehicle (ZEV) program began offering emissions credits for partial zero emission vehicles (PZEV). Over the past 14 years, this has resulted in the sales of millions of gasoline-fueled PZEVs in California and 11 other USA states. Dr. Kubsh characterized PZEVs as gasoline-fueled vehicles that combine the best available three-way catalyst technology with the best available evaporative emission control technology. PZEV technology will also be significantly expanding throughout the USA light-duty vehicle fleet as part of the new USA Federal Tier 3 and California LEV III light-duty vehicle emissions standards. Once these new emissions standards are fully implemented, there will be essentially no contribution to secondary organic aerosol from light-duty vehicles. According to Dr. Kubsh, evaporative emissions standards in Europe are considerably less stringent and lag approximately 20 years behind those in the USA and thus would still be a significant contributor to secondary organic aerosol formation.

According to Dr. Kubsh, the use of GDI is expected to largely supplant the use of port fuel injection (PFI) in gasoline-fueled light-duty vehicle applications. The use of GDI engines in light-duty vehicle applications is increasing worldwide as a means of reducing CO_2_ emissions, largely in response to new regulations reducing emissions of CO_2_ and other emissions that contribute to climate change. Until recently in the EU, the 5 mg/km mass-based PM standard has been very easily met by PFI gasoline engines, which typically have PM emissions at 1 mg/km or less. Second-generation GDI engines (current and recent vehicles) have also been capable of meeting the EU 5 mg/km PM standards. The potential for increased PM emissions from GDI relative to previous PFI engines has resulted in new particulate emissions standards that include both EU6c PN standards in Europe and Federal Tier 3/CARB LEV III PM standards in the USA. The new EU6c particle number (PN) limit of 6 × 10^11^ particles/km is particularly challenging for GDI engines. Both Dr. Kubsh and Paul Whitaker of the AVL Powertrain Engineering, Inc., characterized three options for reducing GDI particulate number emissions:

1. Use of a GPF. This is a wall-flow catalyst capable of filtering and oxidizing exhaust particles similar in many ways to the DPFs used in diesel applications.
2. Use of PFI in addition to GDI. This allows all or some of the fuel wall film to stay outside of the cylinder, particularly during cold start and initial warm-up operation.
3. Refinement of the combustion system, mixture formation and engine control and calibration.

Kubsh discussed the application of GPFs to light-duty vehicles in detail. The systems are robust and work is underway to reduce overall catalyst system cost by integrating three-way catalyst functionality within GPFs to allow them to take the place of conventional under-floor three-way catalysts used for gaseous criteria pollutant control.

**Figure 3:**
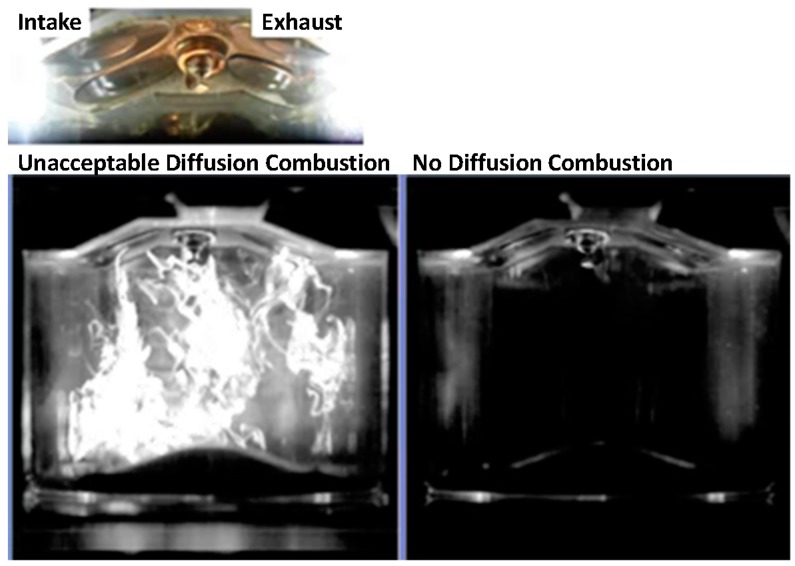
Left-cold piston, high-flame-intensity diffusional combustion is visible above the top of the piston crown due to surface wetting with fuel. This condition resulted in high particle formation. Right-warm piston, homogenous combustion with low-flame-intensity and low particle formation. The picture on the upper left shows the relative positions of the intake valves, exhaust valves and sparkplug.

The presentation by Mr. Whitaker focused on combustion and control strategies to reduce particle emissions from light-duty vehicles equipped with GDI engines. Whitaker described in detail the use of combustion system refinements, improvements in air/fuel mixture formation, and improvements in engine management systems and control system calibration to reduce particle formation during different phases of engine operation. In-cylinder optical measurements have been used to characterize particle formation during engine operation. Conditions with high flame luminance indicate the presence of non-homogeneous, diffusion-limited combustion associated with soot pyrolysis and particle formation (see Figure 3). Methods identified to reduce diffusion-limited combustion in GDI engine applications include:

- Reducing fuel impingement onto surfaces via changes in injector spray targeting, piston bowl shape, injection event timing and use of multiple injections per combustion cycle.
- Changes to spark timing and injection events to directly heat the piston following cold startup to improve the vaporization of impinged fuel.
- Changes to the catalyst heat-up strategy after cold start, including further optimization of the timing and duration of multiple injection events.

Eight recent engine development programs conducted by AVL Powertrain Engineering, Inc. began with PN emissions at up to 6× the EU6c standards and were reduced to 15% to 45% of the EU6c PN standards using such combustion refinements. Mr. Whitaker pointed out that when taking into consideration the need to meet particle emission standards over the full regulatory useful life of a vehicle (e.g., 150,000 miles in California), robustness of system components play a significant role in PM and PN emissions. Optical data showed that injector tip deposits can cause high-intensity diffusion flames near the injector tip from combustion of fuel soaked into injector tip soot deposits, resulting in high PN emissions.

### 7.3. Stationary Source UFP

Professor Pratim Biswas from the Washington University in St. Louis (WUStL) provided a summary of particle emissions from large, coal-fired power plants used for base-load electric generation. A typical coal-fired power plant burns approximately 200,000 kg of coal per hour consisting of coal particles approximately 50 μm in diameter. Prof. Biswas presented results of combustion and emissions studies using both a single-coal-particle drop-tube furnace and 1 MW pilot scale combustion system. Depending on combustion conditions and coal composition, a variety of hydrocarbon species can be emitted including nonaromatic hydrocarbons, aromatic hydrocarbons and carboxylic acids. Oxygenated hydrocarbon compounds are also emitted, particularly from biomass combustion but also to some extent from coal combustion. The contribution of hydrocarbon compounds to PM mass from coal combustion is relatively low when compared to ash or elemental carbon PM emissions but such organic compounds can be important contributors to both primary UFP emissions and secondary UFP formation. 

While black carbon is by far the primary PM mass constituent, Prof. Biswas concluded that it is not a significant contributor to UFP from coal combustion. “Brown” carbon and organic carbon compounds appear to be the primary contributors to UFP from coal-fired power plants and ash formation appears to play a significant role in the formation of these organic aerosols. Inorganic ash compounds can shield organic compounds from oxidation, thus increasing their likelihood of emission. 

O_2_ enrichment of combustion (e.g., oxy-coal combustion) is an important emerging technology for increasing coal combustion efficiency. As O_2_ concentration is increased during combustion, the combustion temperature is elevated and Prof. Biswas indicated that this can lead to increased volatilization of metal oxides, and both higher mass emissions of submicrometer particles and shifts in the size distribution of agglomeration mode particles to larger particle sizes. 

**Figure 4:**
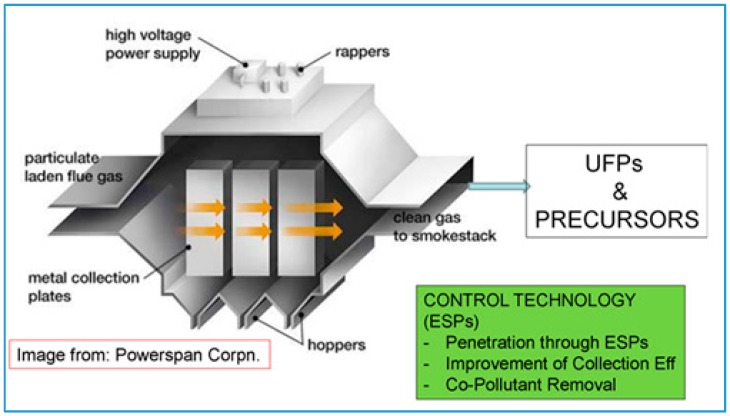
Example of an electrostatic precipitator (ESP) used for controlling PM emissions from stationary sources (courtesy of Prof. Pratim Biswas, Washington University in St. Louis and Powerspan).

The primary PM control technology for stationary combustion is the use of electrostatic precipitators (ESPs). ESPs use an electric field to collect particles from flue gas (Figure 4). Prof. Biswas found that only a small fraction of sub-30 nm particles carry a charge and it is difficult to externally charge sub-30 nm particles. As a result, UFP penetration of sub-30 nm particles through a typical ESP system is relatively high. A better understanding of the charging characteristics of UFP may allow design enhancements to ESPs to improve UFP collection efficiency. Prof. Biswas is investigating the use of a soft X-ray source interacting with the ESP corona discharge to enhance UFP charging and UFP capture efficiency for particle diameters in the 10 nm to 30 nm range. 

## 8. Policy Considerations

The UFP workshop concluded with a discussion of issues related to translating the available scientific evidence to inform policy considerations in the USA and Switzerland. More specifically, presenters provided an overview of evaluations and activities related to: (1) the most recent review of the USA PM NAAQS; (2) California’s progress towards attaining the PM NAAQS and related black carbon (BC) reductions; and (3) a proposal currently under consideration to modify the current Swiss PM standards. As summarized below, a robust discussion followed these presentations primarily focused on potential challenges faced from a science policy perspective and plans for future work. 

Under section 109 of the USA Clean Air Act (CAA), the USA EPA has established NAAQS for PM using PM_2.5_ as the indicator for fine particles and PM_10_ as the indicator for thoracic coarse particles (PM_10–2.5_). In general terms, PM_2.5_ represents particulate matter with an aerodynamic diameter less than or equal to a nominal 2.5 µm and PM_10_ represents particulate matter with an aerodynamic diameter less than or equal to a nominal 10 µm. In regulatory terms, methods for measuring ambient PM_2.5_ and PM_10_ concentrations have been designated as “reference methods” and “equivalent methods” [14]. 

The CAA requires USA EPA to periodically review the science upon which the standards are based and the standards themselves. Scott Jenkins, from the USA EPA, Office of Air and Radiation, provided an overview of the statutory framework for the NAAQS, summarized the history of the PM NAAQS, and briefly discussed the rationale for final decisions reached in the most recent PM NAAQS review completed in December 2012. Specifically, related to consideration of the available health evidence and air quality data for UFPs, the Agency concluded that this information was too limited to provide support for consideration of a distinct standard for UFPs and decided to retain the PM_2.5_ mass-based indicator to continue to provide protection for particles with aerodynamic diameters generally less than or equal to 2.5 micrometers in diameter. The EPA recently initiated its next review of the PM NAAQS and will consider whether the current standards provide requisite public health and welfare protection. If the available information calls into question the adequacy of the current standards, the Agency will consider what alternative standards are supported by the science in terms of the four basic elements of the standards—indicator, averaging time, level, and form. 

California is making remarkable progress in reducing fine particle pollution as it works towards attainment of the NAAQS in the nation’s most challenging PM non-attainment regions. Alberto Ayala from the CARB summarized this state’s success in achieving substantial PM controls while also yielding important and corresponding climate co-benefits by reducing BC. Ayala noted that statewide concentrations of BC have been cut in half from 1989 to 2008. California’s policies have been informed by a portfolio of wide-ranging in-house and extramural research. Over the last several decades, CARB has supported the collection and consideration of a vast amount of data on UFPs from a broad range of scientific disciplines including: metrology, ambient monitoring, modeling, health effects, source attribution, emission control technology, and engine and fuel effects information. In particular, Ayala noted that the anthropogenic sources of UFPs (e.g., stationary, mobile, industrial, and occupational sources as well as atmospheric conversion) are numerous and diverse. CARB has investigated extensively the particle size distribution and total particle number in the emissions from a multitude of sources. Ayala discussed how CARB has participated actively in academic and policy discussions evaluating the strengths and limitations of various UFP metrics (e.g., mass, particle number, particle surface area). He specifically highlighted the achievements of the California light-duty vehicle tailpipe PM standards (e.g., LEV III) and challenges in controlling aircraft emissions and evaluating potential indoor exposures. 

The Swiss Federal Commission for Air Hygiene (FCAH) advices the Swiss Government on the regulation and air quality standards of ambient air pollution to protect public health. FCAH recently reviewed the current Swiss PM standards in light of the availably scientific evidence. The President of the FCAH and Deputy Director of the Swiss Tropical and Public Health Institute (Swiss TPH), Nino Künzli, provided an overview of the Swiss perspective of PM standards. Specifically, Künzli summarized the rationale supporting FCAH’s recent proposal to adopt the World Health Organization (WHO) annual PM_2.5_ standard, for which Switzerland does not have standards yet. With regard to UFPs, Künzli articulated the reasons FCAH has abstained from proposing a distinct air quality standard for UFP particularly focused on the lack of standards for monitoring UFPs/particle number. The FCAH proposes rigorous reductions in elemental carbon (EC) emissions—a marker of fine and ultrafine combustion-related particles—using the best available technology. If this policy is adopted, Switzerland estimates that the ambient EC concentrations will be reduced, at all sites, by 80%. In addition, monitoring of UFPs, EC, and other near-source pollutants was proposed. The FCAH proposal is currently under evaluation as the Swiss Government and Cantonal agencies discuss its implications on monitoring. A public evaluation will follow in 2015 prior to the Government making final decisions. Künzli encouraged international standardization of measurements and expanded ambient monitoring to improve our understanding of UFPs and other pollutants (e.g., EC, OC).

The discussion that followed the panelists’ presentations emphasized that the methods and metrics for identifying and characterizing emissions and impacts from UFP exposures have not been consistent. As a result, integrating information across studies has been difficult and, thus, it is challenging to adequately assess the potential health effects attributed to UFPs that are separate and distinct from those associated with PM_2.5_ and other co-pollutants within the broader ambient mixture to inform policy decisions.

## 9. Consideration of Next Steps

Workshop participants strongly supported an ongoing dialogue to promote international collaborations and information exchange to improve our understanding of UFPs across scientific disciplines. More specifically, participants generally supported efforts to promote standardization of measurements and metrics while continuing to expand the monitoring of UFPs and related pollutants (e.g., BC) to better characterize air quality trends and potential exposures, especially in near-source environments. As an initial step, participants discussed the need to identify the strengths and limitations of the available UFP metrics and to explore options for developing consensus for a metric(s) that will integrate emission and ambient measurements with future exposure and health studies. In evaluating epidemiological evidence, participants stressed the importance of understanding the role of UFPs within the broader ambient mixture; specifically, differentiating effects associated with UFP exposures from effects associated with PM_2.5_ or other co-pollutants.
